# Secret Sarcoma: A Cardiac Mass Disguised as Influenza

**DOI:** 10.1155/2018/8628365

**Published:** 2018-06-06

**Authors:** Christopher DeZorzi, Katherine I. Harris

**Affiliations:** University of Iowa Hospitals and Clinics, Iowa City, IA, USA

## Abstract

This case presentation discusses an extremely rare diagnosis presenting with common symptoms, attributed to influenza, which were not investigated further when clear cardiac symptoms developed. The patient initially presented with symptoms consistent with influenza, but when orthopnea and dyspnea on exertion developed, these cardiac symptoms continued to be attributed to a postviral syndrome and were not further evaluated. Premature closure bias contributed to a delay in diagnosing a rare cardiac condition. The diagnostic momentum, or the continuing of a diagnostic label, occurred across multiple providers and settings. This case demonstrates the risk of premature closure and diagnostic momentum and reminds clinicians to reframe the differential diagnosis as more information on history or physical exam becomes available.

## 1. Introduction

A cardiac sarcoma is a rare malignant cardiac tumor that can present with nonspecific symptoms (similar to most cancers) in combination with clinical signs of heart failure due to the mass effect of the tumor. Timely diagnosis is crucial to prevent further obstruction as the tumor grows. If the tumor is recognized in the earlier stages, complications including heart failure, arrhythmias possibly leading to sudden cardiac death, and ischemia secondary to obstruction of coronary arteries can be avoided. Additionally, early detection can allow treatment before the cancer can metastasize.

## 2. Case Presentation

A 50-year-old Asian male with a past medical history of supraventricular tachycardia and obstructive sleep apnea on CPAP at night presented with one month of intermittent flu-like symptoms, orthopnea, and dyspnea on exertion. At the onset of these symptoms, he presented to a walk-in clinic and was diagnosed with influenza. He was treated symptomatically and noted improvement, but one week later he had a recurrence of symptoms while playing volleyball. From that time on, he noticed dyspnea on exertion, continued malaise, fevers, and diffuse joint pains so he presented multiple times to outpatient providers. He received doxycycline without improvement, and follow-up testing showed a mild leukocytosis, negative EBV, and an unremarkable chest X-ray. He was diagnosed with lingering postviral symptoms from influenza. He ultimately presented as a walk-in patient to the cardiology clinic when he started having chest tightness, palpitations, and his dyspnea progressed to occurring at rest, relieved only with a tripod position.

**Figure 1:**
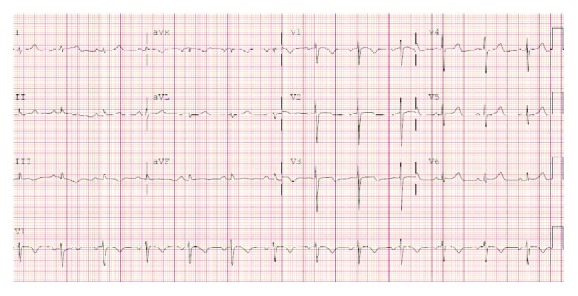
EKG on presentation showing right axis deviation and ST-T wave changes.

**Figure 2:**
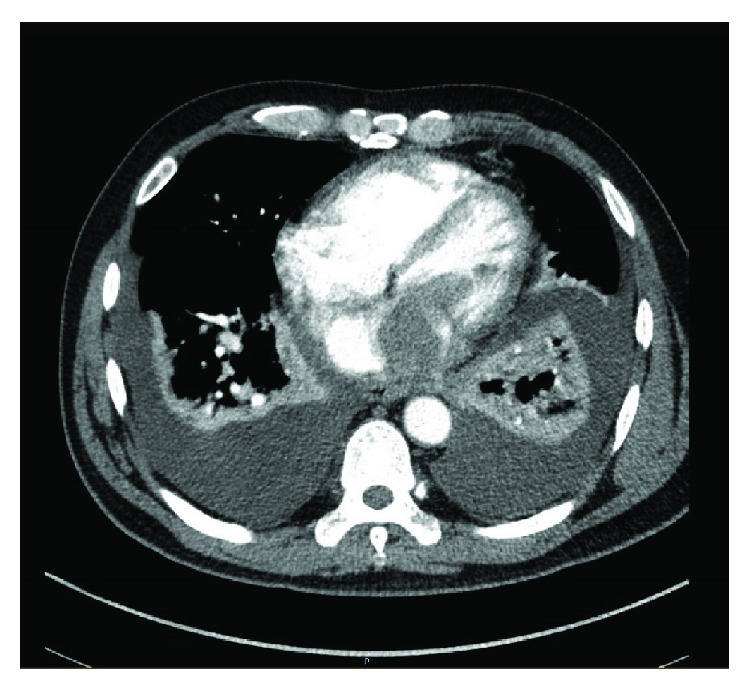
CT angiogram displayed moderate bilateral pleural effusions, a mass in the right ventricle, and a mass invading into the left atrium through the mitral valve into the left ventricle.

**Figure 3:**
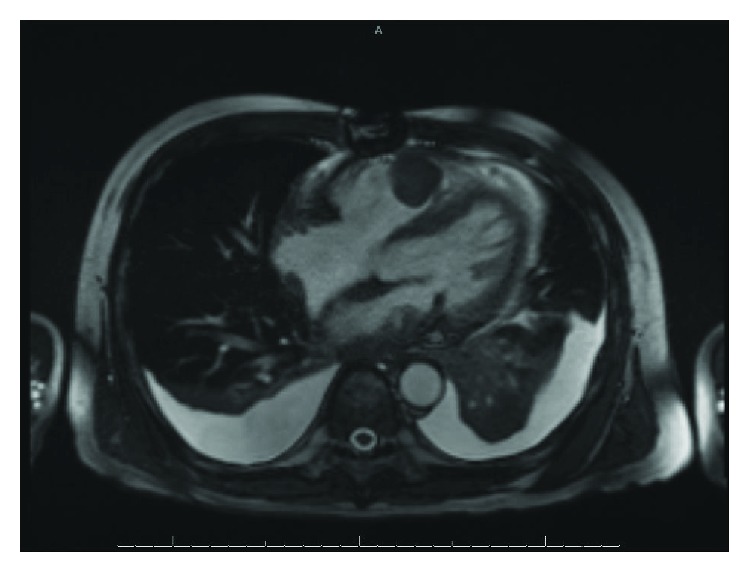
MRI confirmed masses in the right and left ventricle. Shown above is an image of the mass located in the right ventricle.

EKG on presentation (Figure 1) showed right axis deviation and abnormal ST-T wave segments in V1 through V3 which was new compared with a prior EKG. Due to the concern for pulmonary embolism, a CT angiogram of the chest was obtained which displayed moderate bilateral pleural effusions, a mass in the right ventricle, and a mass in the left atrium extending through the mitral valve invading into the left ventricle (Figure 2). Echocardiogram exhibited normal LVEF but some mitral valve occlusion due to the mass. Cardiac MRI was obtained (Figure 3) and confirmed the masses. The patient required debulking of the left atrial tumor, and pathology revealed an undifferentiated, high-grade pleomorphic sarcoma. Due to tumor infiltration into the left pulmonary veins, as well as focal areas of uptake in the small bowel at a site of intussusception, he was started on pembrolizumab chemotherapy with concurrent radiation therapy to the heart and small bowel.

## 3. Discussion

A cardiac sarcoma is a primary malignant cardiac tumor. These are exceedingly rare, with the incidence of primary neoplasms of the heart found at autopsy being 0.017%–0.056% [1, 3]. Of these rare primary cardiac tumors, up to 75% are benign and are usually myxomas, lipomas, or rhabdomyomas. Only 25% of primary cardiac tumors are malignant and, of these, 75% are sarcomas [4]. Tumors presenting in the heart are more frequently secondary tumors than primary. The incidence of secondary tumors is 1.23% with the three most common malignant neoplasms to the heart being from lung cancer, esophageal carcinoma, and lymphoma. Risks of a cardiac sarcoma include malignant spread and physiologic effects on the heart including compression of the coronary arteries or pericardial space, arrhythmias, and outflow obstruction. Sudden death is associated with primary neoplasms approximately 0.0025% of the time. Although 86% of these were classified as benign, their locations within the heart lead to conductive and hemodynamic abnormalities resulting in sudden death [2]. In previous studies, most patients with a cardiac sarcoma were dead within one year of their diagnoses [5] and had a median survival time of 6 months [6]. In a newer study, patients who underwent resection with curative intent and survived surgery (surgical mortality was 7.4%), the median survival was 23.5 months [8]. Nonsurgical options for treatment of cardiac sarcomas can be difficult due to radiation effects on the surrounding heart and the high levels of radiation (6000–6500 cGy) that is typically needed. Also, chemotherapy effective against sarcomas, such as Adriamycin, is typically avoided due to its cardiotoxic side effect profile [7].

The epidemiology indicates the rarity of this case, but the importance of recognizing this presentation in an outpatient setting before it progresses should not be lost. Beyond adding to the differential diagnoses of a patient presenting with progressive dyspnea on exertion, this case reinforces the importance of reframing the diagnosis when seeing a patient multiple times within a short timeframe. This patient was seen four times within a month by a combination of outpatient providers. Premature diagnostic closure bias creates difficulties for busy practitioners to reassess a case and develop a new differential when the previous diagnosis no longer applies. Our patient had a viral-like illness at the onset, but persistent symptoms of dyspnea on exertion and orthopnea should prompt a cardiac workup. An EKG showing right axis deviation and ST-T wave changes may have alerted practitioners that further workup is needed, and an echocardiogram or CT angiogram would have discovered the rare underlying diagnosis and allowed for expedited diagnosis and treatment.
